# Vibrotactile enhancement of musical engagement

**DOI:** 10.1038/s41598-024-57961-8

**Published:** 2024-04-02

**Authors:** Kai Siedenburg, Michel Bürgel, Elif Özgür, Christoph Scheicht, Stephan Töpken

**Affiliations:** 1 Graz University of Technology, Signal Processing and Speech Communication Laboratory, 8010 Graz, Austria; 2 Department of Medical Physics and Acoustics, Carl von Ossietzy Universität Oldenburg, 26129 Oldenburg, Germany

**Keywords:** Human behaviour, Engineering

## Abstract

Sound is sensed by the ear but can also be felt on the skin, by means of vibrotactile stimulation. Only little research has addressed perceptual implications of vibrotactile stimulation in the realm of music. Here, we studied which perceptual dimensions of music listening are affected by vibrotactile stimulation and whether the spatial segregation of vibrations improves vibrotactile stimulation. Forty-one listeners were presented with vibrotactile stimuli via a chair’s surfaces (left and right arm rests, back rest, seat) in addition to music presented over headphones. Vibrations for each surface were derived from individual tracks of the music (multi condition) or conjointly by a mono-rendering, in addition to incongruent and headphones-only conditions. Listeners evaluated unknown music from popular genres according to valence, arousal, groove, the feeling of being part of a live performance, the feeling of being part of the music, and liking. Results indicated that the multi- and mono vibration conditions robustly enhanced the nature of the musical experience compared to listening via headphones alone. Vibrotactile enhancement was strong in the latent dimension of ‘musical engagement’, encompassing the sense of being a part of the music, arousal, and groove. These findings highlight the potential of vibrotactile cues for creating intensive musical experiences.

## Introduction

Music can be heard through the ears and felt through the body via acoustic and vibrotactile sensory receptors, respectively. A variety of approaches for the vibrotactile enhancement of music have been researched in recent years^1–5^, see Paisa et al. for a review^6^. Despite these advances, rigorous evaluations of the effects of vibrotactile stimulation on music perception are still scarce. Consequently, the aspects or latent dimensions of musical experience that are affected most pertinently by vibrotactile stimulation remain unclear. The goal of the present work is to contribute to a deeper understanding of the perception of audio-tactile stimuli in the realm of music, which is an essential step for developing new audio-tactile devices tailored to normal-hearing and hearing-impaired listeners and for exploring applications of vibrotactile stimulation in music therapy.

When events in the world stimulate separate sensory modalities, the brain often exploits the congruence of sensory signals via multisensory integration^7^. A possible breeding ground for multisensory integration are robust structural correlations between stimulus attributes of different sensory modalities. Vibrotactile and acoustic stimuli are often structurally correlated, because bass frequencies directly affect the quality of the music experience via whole-body vibration at high sound levels^8^–imagine concerts of pop/rock music or the sound of low organ pipes. It is long known that vibrotactile and auditory perception share important qualitative features, such as frequency dependent detection thresholds, frequency-based masking, energy integration over time, and temporal gap detection^9^. Almost 70 years ago, van Bekesy already wrote on the striking commonalities between the ways in which the skin and the cochlear transform vibration into nerve signals^10^. At the same time, concrete psychophysical quantities such as detection thresholds or time constants are very different in vibrotactile and auditory perception. The fidelity of hearing surpasses vibration perception in many respects, as in the case of frequency bandwidth, dynamic range, or frequency resolution^9^.

More recent research has also identified clear enhancement effects when vibrotactile stimulation complements auditory perception. This includes clear and long-studied effects of congruent vibrotactile enhancement of auditory loudness perception^11,12^ and audio-tactile integration of rhythm^13^ and roughness^14^ perception. Furthermore, research has indicated strong effects of congruent vibrotactile stimulation on auditory localization^15^ and speech understanding^16,17^ for listeners with cochlear implants (CI). In the latter study, Fletcher and colleagues derived vibrotactile signals directly from the audio signal, which were then presented as a form of haptic augmentation of the acoustic stimuli. Concerning pitch perception, spatio-spectral mappings of acoustic to tactile cues were explored that allowed to use skin location and vibrotactile frequency cues^18^, demonstrating enhanced pitch perception of CI listeners, which is a central problem for music perception of CI listeners. The potential for training regimes to improve vibrotactile pitch discrimination has also been addressed recently^19^. With regards to timbre, Russo et al.^20^ demonstrated that vibrotactile stimulation allows participants to successfully discriminate sounds with dull and bright timbre, suggesting frequency-tuned mechanoreceptors functioning as critical bands. Reseachers have also studied crossmodal effects of vibrotactile stimulation on timbre dissimilarity ratings of isolated synthetic sounds^21^. Specifically, the dimensions of a dissimilarity space derived from the ratings of normal-hearing listeners could be accurately modeled by a linear combination of audio and tactile stimulus features (attack time, roughness). Using simple sine wave melodies, it was further observed that time and intensity alignment are most critical for ratings of stimulus preference (used as a proxy of vibrotactile coherence)^22^. Frequency congruence was only observed to play a notable role for musician participants with frequency changes stretched by a factor of 1.5, suggesting a negligible role in terms of practical relevance. With regards to more general aspects of musical experience, studies have also shown that vibrotactile stimulation congruent with the music improves ratings of groove and enjoyment^23^, and that dance behavior can be affected by auditorily undetectable, very low frequency sound^24^.

Given that previous research has observed salient effects of multisensory integration between acoustic and vibrotactile stimuli, the development of audio-tactile interfaces appears to be a promising path for enhancing music perception, potentially even for listeners with a sensory impairment and/or CI. However, previous research is limited in four important respects in that it has been (i) predominantly working with artificial stimuli, (ii) concentrating on stimulating the whole body or only small skin surface areas of single body parts with one vibration signal, (iii) evaluating the resulting perceptual dimensions with a relatively narrow set of dependent variables, and (iv) not considering the potential role of individual differences in audio-tactile integration. Consequently, there remains a gap in our understanding of the perceptual implications of vibrotactile music enhancement in realistic situations. With the present research, we aimed to go beyond the noted limitations of the literature to study how latent factors underlying musical experience are affected by vibrotactile stimulation. This aim was approached by using (i) realistic musical stimuli, (ii) an audio-tactile interface that allowed us to provide a multi-source vibrotactile display, and (iii) a relatively broad set of evaluation scales. In addition, (iv) we considered an important predictor of individual differences in auditory tasks, namely musical sophistication and its association with rating behavior.

We presented excerpts of multi-track music (unknown royalty-free tracks from popular genres) to young normal-hearing participants and had them pro rate six items, covering a wide range of important aspects of musical experience. Specifically, we asked (1) how pleasant participants found the effect of the music (*Valence*), (2) how energetic/arousing they found the effect of the music (*Arousal*), (3) how much they wanted to move to the groove of the music (*Groove*), (4) how much they felt like being part of a live performance of the music (*Live-Feeling*), (5) how much they had the feeling of being part of the music (*BeingPart*), and (6) how much they liked the music (*Liking*). The valence and arousal items were selected to cover emotional experiences with music and previous research had shown the adequacy of the two-dimensional model^25^. The groove item was chosen to cover the important facet of sensorimotor coupling to music^26^. Whether or not music feels as if performed live was deemed an important aspect of musical experience, given the elevated status that life concerts still possess in musical culture^27^. The item on the feeling of being part of the music assesses whether the experience has an enveloping effect, analogous to studies on the immersive effects of virtual acoustical reality^28,29^. Finally, the liking scale measured the overall preference of an experimental condition, important for an overall assessment of the adequacy of a condition.

**Figure 1:**
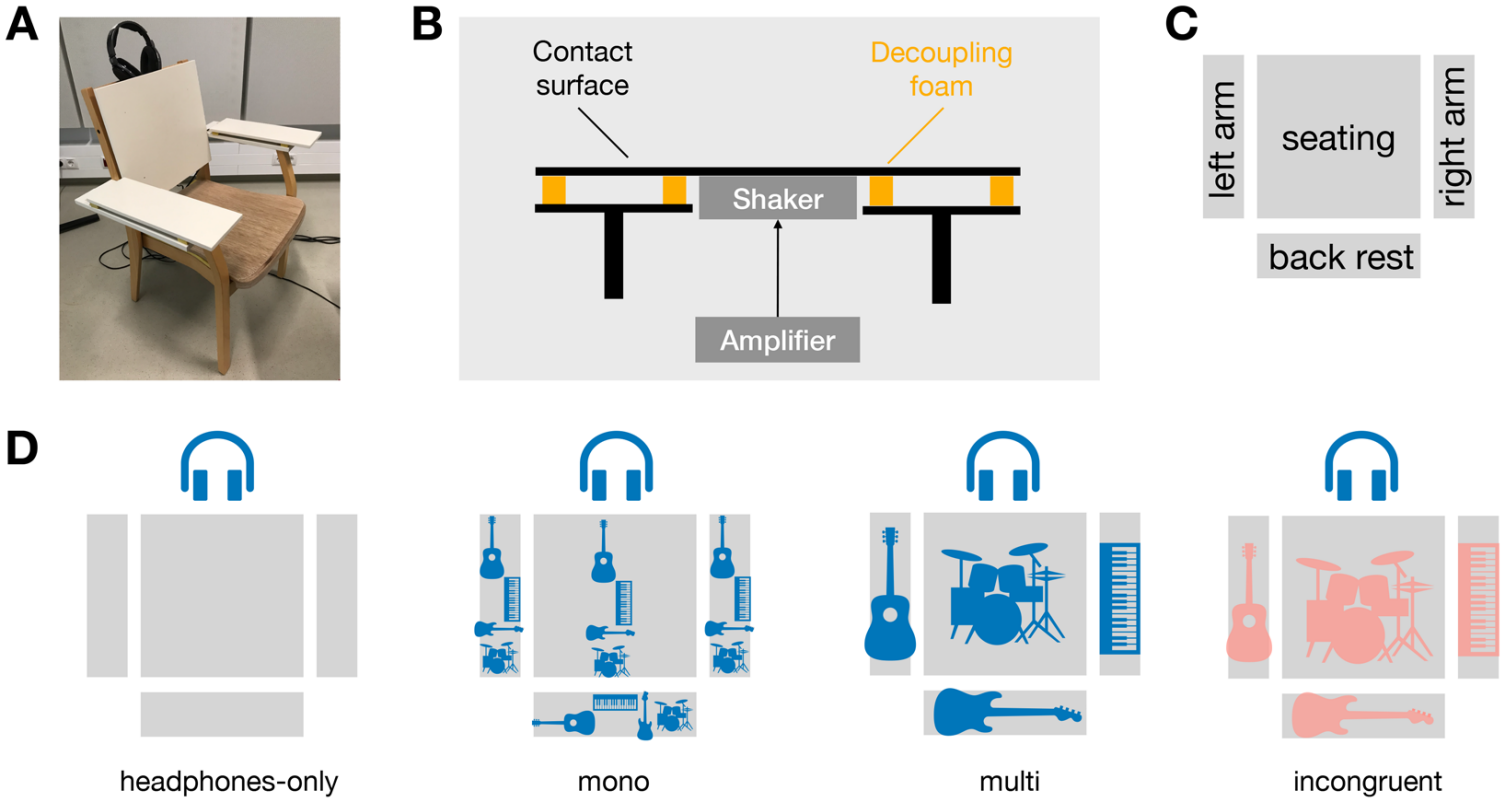
(**A**) Participants were seated on a chair through which the vibration stimulus was presented. The acoustical stimulus was presented via headphones. (**B**) Electro-dynamic shakers were attached to the opposite sides of the contact surfaces of the chair. Contact surfaces were mechanically decoupled from the chair frame and each other. (**C**) There were four contact surfaces. (**D**) Schematic of the vibration conditions. No vibration was presented in the headphones-only condition, the same low-pass filtered mono rendering of the audio was presented to all contact surfaces in the mono condition, vibration signals from different instruments were presented to different contact surfaces in the multi condition, and vibration signals from a completely different audio example were presented in the incongruent condition, indicated by different colors of headphone and vibration symbols.

Participants were seated on a custom-made chair and vibrotactile stimuli were presented via its four mechanically decoupled contact surfaces (Fig. 1A–C). A chair is highly suited as a vibrotactile interface, since it can provide a multi-source display of the multi-channel audio while allowing listeners to take a naturalistic body posture during listening. This approach let us compare how listeners rate a multi-source display of vibrations in comparison to vibrations driven by a mono rendering of the audio signal, no vibrations at all, or vibrations incongruent with the audio signal. We hypothesized that multi-track vibration would yield the most salient effects of music evaluation ratings compared to the headphones-only condition with no vibration at all. On the contrary, we hypothesized that incongruent vibration stimuli would be perceived as most unpleasant compared to all other conditions.

## Results

**Figure 2:**
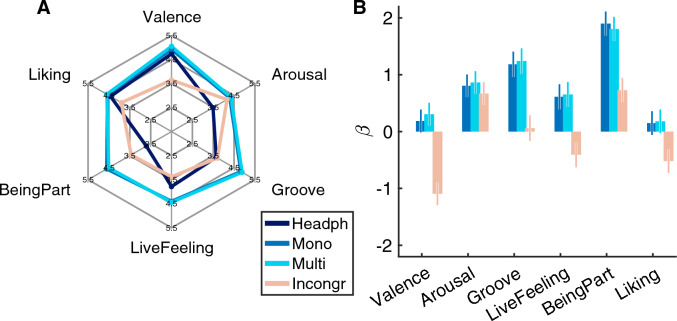
(**A**) Spider plot of group averages for the six different items. (**B**) Estimated coefficients and confidence intervals of a linear mixed-effects (LME) model with the headphones-only condition used as reference.

The experiment was carried out with 41 participants in total, which were tested in two cohorts. Cohort 1 consisted of 22 participants and Cohort 2 consisted of 19 participants. The test setup was identical for both cohorts, although a few details regarding the stimulus presentation differed (see Methods). Despite these differences, ratings from the two cohorts of participants were almost identical, with an extremely high correlation between their average profiles, $r(23) = 0.997, p <0.001$. For that reason, the data from the two cohorts are jointly analysed and reported in the following. Averages and 95% confidence intervals of ratings are shown in Table 1. Results are further visualized in Fig. 2A, using a spider plot to depict the mean profile of the vibration conditions across the six items. Whereas the headphones-only and the incongruent condition yielded very different shapes, the mono and multi conditions clearly maximized ratings along all scales and were very similar to each other.

The strength of the effects of the vibration conditions are further visualized in Fig. 2B. The figure shows the estimated fixed effects coefficients from a linear mixed-effects model for every item with random intercepts for participants and songs, using reference-based dummy coding (with headphones-only as reference condition). The estimated $\beta$-coefficients may thus be understood as a measure of effect size and direction. According to these estimates, it is clear that incongruent vibration signals had a clearly detrimental effect on valence. Consistently, the incongruent stimulus condition was also the least liked compared to all others. Furthermore, any type of additional vibration increased the arousal ratings compared to the headphone only baseline. The addition of a vibratory stimulation increased the feeling of being a part of the music even more than arousal. Surprisingly, the feeling of being a part even increased for an incongruent stimulus compared to the headphone only baseline.

**Table 1:** Mean values for all conditions with 95% confidence intervals in brackets.

	Valence	Arousal	Groove	Live feeling	Being part	Liking
Headph	4.7 [4.6, 4.9]	3.5 [3.3, 3.7]	3.6 [3.3, 3.9]	3.8 [3.5, 4.1]	2.7 [2.4, 3.0]	4.4 [4.2, 4.7]
Mono	4.9 [4.7, 5.1]	4.3 [4.1, 4.5]	4.8 [4.6, 5.1]	4.4 [4.1, 4.7]	4.6 [4.2, 4.9]	4.6 [4.3, 4.8]
Multi	5.0 [4.9, 5.2]	4.4 [4.2, 4.5]	4.9 [4.7, 5.1]	4.4 [4.1, 4.7]	4.5 [4.2, 4.8]	4.6 [4.4, 4.8]
Incongr	3.6 [3.4, 3.9]	4.2 [3.9, 4.4]	3.7 [3.4, 4.0]	3.4 [3.0, 3.8]	3.4 [3.1, 3.8]	3.9 [3.6, 4.2]

Rows refer to stimulus conditions, columns to dependent variables (items).

Because the scales on which ratings were provided were highly correlated (only two of the 15 correlations between ratings of the six items were insignificant at level of $p>0.001$), the possibility of an underlying latent structure was considered. To investigate such a latent structure, an explorative principal components analysis (PCA) was computed on the means of every vibration condition and participant (i.e., on a 164 × 6 matrix, due to 41 participants, 4 vibration conditions, and 6 scales). A highly significant Bartlett’s test of sphericity indicated suitability for factor analysis ($\chi ^2(15) = 570, p <0.001$).

**Figure 3:**
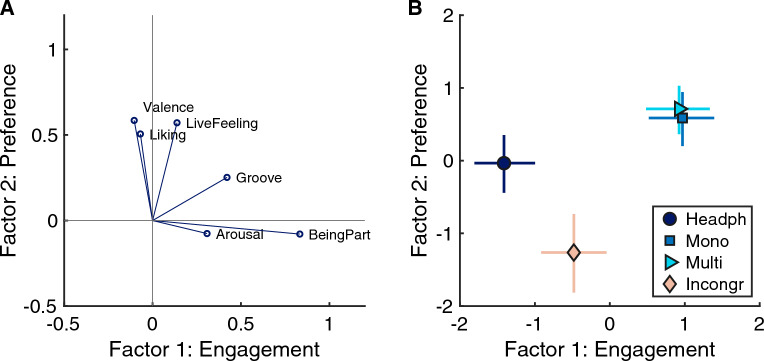
(**A**) Positions of the six rating scales in the two-dimensional factor space. Factor 1 is interpreted as corresponding to *engagement*, Factor 2 (after sign reversal) to *preference*. (**B**) Factor scores for the four vibration conditions. Cross corresponds to group average and 95% CIs along the two dimensions.

The first two resulting factors accounted for 81.6% of the variance in the data (Factor 1: 64.5%, Factor 2: 17.2%) with a clear knee point in the scree plot. For that reason, a two-dimensional projection was adopted. To increase ease of interpretation, components were rotated using a Varimax rotation and the resulting factors are displayed in Fig. 3A. The items BeingPart, Groove, and Arousal (in descending order) loaded strongly on the first factor, whereas the second factor received strong loads of the items Valence, Live-Feeling, and Liking (in descending order). The first factor was therefore interpreted as reflecting a sense of musical *engagement*. The second factor appeared to be associated with aversion (i.e., opposite of preference); yet for the sake of simplicity, we reversed the sign of the solution such that the factor could indeed be interpreted as a (positive) measure of *preference* in Fig. 3. The orientation of the used rating scales in the two-dimensional space are displayed in Fig. 3A; the average factor scores across participants for the four vibro-acoustic conditions are displayed in Fig. 3B.

Although there is considerable spread in terms of individual responses along the two factors, group means were robustly separated for all vibro conditions except the mono and multi conditions. Along the engagement dimension, the headphones-only condition had the lowest scores (M = − 1.4 with 95% confidence interval [− 1.8, − 1.0]), followed by the incongruent condition (M = − 0.48 [− 0.89, − 0.03]), and the very similar mono (M = 0.97 [0.52, 1.4]) and multi conditions (M = 0.92 [0.44, 1.3]) with largely overlapping confidence intervals. Along the preference dimension, the incongruent condition had lowest scores (M = − 1.3 [− 1.8, − 0.7]), followed by the headphones only condition (M = − 0.03 [− 0.4, − 0.37]), and once again the mono (M = 0.59 [0.23, 0.94]) and multi conditions (M = 0.71 [0.39, 1.0]) with very similar scores and overlapping confidence intervals. Paired t-tests confirmed that the means of the mono and multi conditions were statistically indistinguishable along both dimensions ($p > 0.20$). Yet, both conditions had significantly higher engagement scores compared to the headphones-only condition ($t(40)>8.2, p <0.001, d > 1.3$). Mono and multi conditions also had higher preference scores compared to the headphone-only condition ($t(40)> 3.4, p <0.002, d > 0.53$). Along both dimensions, the scores of the incongruent condition were significantly reduced compared to the those of the mono and multi conditions ($t(40)= -5.2, p < 0.001, d > -0.82$). The means of the incongruent condition also differed from the headphones-only condition along both dimensions ($|t(40)|> 5.1, p <0.001, |d| > 0.80$).

In sum, these results suggest that the presence of congruent vibrations increased musical engagement to a considerable extent compared to pure headphone listening with strong effect size ($d > 1.3$), based on an enhanced sense of arousal, groove, and the feeling of being part in the music. Whereas the presence of incongruent vibrations clearly affected preference in a negative way, the preference of a musical excerpt was positively affected by the presence of vibrations, although only with a weak effect size ($d > 0.5$).

**Figure 4:**
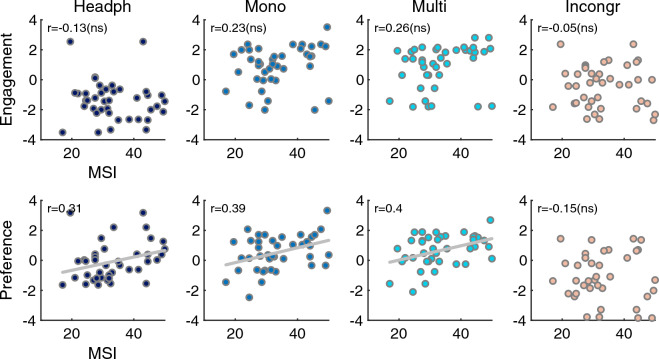
Relationship between the musical sophistication index (MSI) and the Engagement (top row) and Preference factors (bottom row). Lines correspond to linear regression lines between MSI and preference scores in case of significant correlation coefficients.

As indicated by the relatively wide confidence intervals in Fig. 3B, there were non-negligible individual differences in the evaluation of audio-tactile stimuli. Figure 4 shows the relation between participants’ musical sophistication index (average of Gold-MSI training and perception subscales^30^) and participants’ scores in all four vibration conditions. As visible, there were no significant correlation for the engagement factor ($r(40) <0.26, p > 0.10$), indicating that musical sophistication was not associated with how engaging the music was experienced. Yet, we observed a correlation between musical sophistication and preference scores for the headphones-only ($r(40) =0.31, p =0.049$), mono ($r(40) =0.39, p = 0.014$), and multi conditions ($r(40) = 0.40, p = 0.009$), but not for the incongruent condition ($r(40) = -0.14, p = 0.36$). That is, there was a particularly strong association between musical sophistication and preference of the stimuli when congruent vibrations were present, and a less strong association for the headphones-only condition, but there was no association between musical sophistication and preference in the incongruent condition. Participants with higher levels of musical sophistication thus generally provided higher preference scores except for the incongruent condition.

## Discussion

In the present study, we contrasted the effects of four types of vibration conditions on the experience of excerpts of music from popular genres that was probed by an array of six rating scales. Vibrations were presented via the contact surfaces of a chair. Results revealed considerable differences in music evaluation ratings across vibration conditions, with mono and multi conditions yielding very similar patterns of results, both of which tended to boost responses along the rating scales compared to no vibration (headphones-only). Specifically, the feeling of *being part of the music*, together with indices of groove and arousal–that we jointly conceived as a musical engagement latent factor–improved considerably with the presentation of congruent vibrotactile cues. However, preference scores only weakly improved with the addition of vibration signals.

Previous work showed that audiotactile stimuli tend to yield more forceful tapping with the beat of the music, more spontaneous body movement, and higher ratings of groove and enjoyment^23^. The present findings extend this line of work by suggesting that the feeling of groove and sensorimotor synchronization may be considered as part of a more general process of musical engagement that goes along with states of high arousal and the feeling of being part of the music. Particularly the latter could also be framed as a musical form of immersion^31^, that vibrotactile cues appear to have strong effects on. These findings could further imply that the clear increases in overall quality ratings found by Merchel and Altinsoy^8^, when they added whole-body vibrations to loudspeaker playback of classical music, might be explained to some extent by an increased engagement and only to a lesser extent by preference decisions.

Contrary to our hypothesis, there was no clear advantage of the multi over the mono condition in its present implementation. The difference in tactile stimulation between the conditions was clearly noticeable but did not create a marked effect when applied to perceptually rich music signals. Therefore, a puzzle to solve concerns the aspect of multi-source vibration and the extent to which participants can integrate acoustical cues with vibration cues across different body parts. In the present experiment we did not find clear advantages of multi-source vibration that was spatially congruent with the audio stimulus, suggesting a limited capacity for multi-source audio-tactile integration. After all, it seems that the separation of sources in the vibrotactile domain does not affect emotional experience of the music. However, this should be followed up on in future research.

We further observed a correlation between musical sophistication scores (Gold MSI training and perceptual abilities subscales) and preference of vibration in the headphones-only, mono, and multi conditions, with greater correlations in the latter two conditions. Previous research has identified associations between the big-five personality dimension of Openess to Experience and musical sophistication^32^. Consequently, one interpretation of the present correlation could be that differences in personality may be associated with differences in preferences for or against vibration stimuli complementing the music. Additionally, one may point to the association between cognitive resources and musical sophistication^30^, where cross-modal integration between acoustic and vibration stimuli would require cognitive load, thus being preferred by individuals with greater cognitive resources. With the present work, we suggest the relevance of individual differences in audio-tactile integration, but further research is required to tease apart the relevance of specific variables such as age, openess to experience, musical training, etc.

Specific limitations of this study deserve mention. As a means to study audio-tactile perception in a setting of potential real-world impact, we used naturalistic excerpts of unknown multi-track music from popular genres. Due to the wide range of spectro-temporal variability and differences in the average frequency content of the tracks, this approach naturally came along with a lack of control with regards to the vibration stimulus, because we used the low-pass filtered audio signal to directly drive the vibration signal. Recent results (which we were not aware of at the time of designing the experiment) indeed suggest alternative approaches for deriving the vibration stimulus from the audio. Specifically, frequency congruence between acoustic and vibrotactile stimuli appears to play a negligible role for audio-tactile integration^22^ and alternative preprocessing methods have been shown to hardly differ in rated overall quality in the music context^8^. It thus seems promising to explore approaches using a carrier signal which fits to the frequency response of the used shaker system and modulating it with the envelope of the music test signal. Furthermore, we here decided to use one fixed level of vibration in order to be able to interpret individual differences of vibrotactile effects. In future research, it seems valuable to include an experimental condition wherein participants are allowed to individually adjust the preferred intensity of the vibration signals. Finally, it could be valuable to refine and simplify the formulation of some of the rating scales to guarantee accessibility for a broad range of subjects in future research.

An important finding of this study is that vibrotactile stimulation affects different aspects of musical experience in different ways: we found a strong enhancement of variables related to musical engagement, that is, the perception of being part of the music, groove, and arousal. We observed more subtle positive effects of vibrotactile stimulation on variables related to preference of the music. Despite the relative homogeneity in terms of age and educational background of our test subjects, we observed individual differences in engagement and preference scores, likely calling for an individualized treatment of vibration stimuli, if used as a way to augment music perception. Taken together, these results indicate that similar to acoustical hearing aids, audio-tactile devices may require a careful *fitting process*, if used for enhancing music perception.

## Methods

### Participants

Participants were recruited as part of two cohorts: Cohort 1 comprised 22 young participants (M = 25.1, 11 F, 11 M) with self-reported normal hearing. Their degree of musical training was assessed using the Goldsmith Musical Sophistication index^30^. Gold MSI scores of Cohort 1 were M = 41.9 (SD = 5.3) for the Perceptual Abilities subscale and M = 20.6 (SD = 8.6) for the Musical Training subscale. Cohort 2 comprised 19 young, participants (M = 25.4, SD = 2.7 12 F, 7 M) with self-reported normal hearing and Gold MSI scores of M = 47.8 (SD = 9.2) for the Perceptual Abilities subscale and M = 24.2 (SD = 13.6) for the Musical Training subscale. Participants read an information sheet and provided informed consent in writing before the start of the experiment. They were compensated with 12 Euro per hour.

### Stimuli

Acoustical stimuli were generated in MATLAB (MathWorks Inc., Natick, MA, USA) by extracting excerpts from a multitrack music database (“MedleyDB”, https://medleydb.weebly.com/). The database comprised 127 royalty-free songs representing a wide range of popular music genres, with individual audio files for each instrument and vocals. The majority of the songs had English lyrics. In the experiment, eight 120 s-long excerpts were drawn from songs in the database, each containing tracks from the instrument categories bass, drums, guitar, and piano, plus a vocal track. The excerpts were divided into four 30-s segments, yielding one distinct stimulus per song for each of the four vibration conditions. This was done to avoid exact repetitions of the music stimuli across the four vibration conditions and the potentially confounding effects while keeping acoustic parameters of the stimuli relatively balanced. The specific songs and excerpts that were used including the musical beat rate are listed in Table S1 in the Supplementary Materials.

Vibrotactile stimuli were derived from the low-pass filtered audio of the acoustical stimuli and presented via four contact areas of the body (left arm, right arm, back, buttock) with the chair that participants were seated on. Four vibro-tactile stimulation conditions were employed: *headphones-only, mono, multi*, and *incongruent*. In the *headphones-only* condition, no vibro-tactile stimulation was provided, resulting in an exclusively acoustical stimulation. In the *mono* condition, all instrument tracks (excluding the vocals) were presented by all shakers, generating the same vibration signal at each of the four stimulation sites. In the *multi* condition, instrument tracks of the acoustical stimulation were mapped to fixed chair positions: guitar to the left arm rest, piano to the right arm rest, drums to the seat surface, and the bass to the back rest. The lateral mapping was the same as in the stereo mix of the audio signal where the guitar was panned to the left, the piano panned to the right ear while drums and bass were presented in the center (diotically), see below. In the *incongruent* condition, the instrument tracks were assigned to chair positions following the same mapping as in the *multi* condition; however, the vibration signal was derived from a completely different song (randomly selected from the database), leading to an incongruency between acoustical and vibro-tactile stimulation.

### Presentation

The acoustical stimuli were presented as stereo signals through open headphones. The root-mean-square (RMS) sound levels of all tracks were normalized. All tracks were summed up to create the mixture and its sound level was normalized to an average output level of 70 dB SPL(A) at the ear drum. The musical scene was arranged such that the bass, drums, and vocals were panned to the center, whereas the guitar was panned to the left side, and the piano to the right side of the scene. This was achieved by balancing the amplitude of the instruments to be 75% (− 22.5 dBFS) on the dominant side and 25% (− 32 dBFS) on the respective other side, with tracks in the center distributed evenly with 50% (− 26 dBFS) on both sides.

The vibrotactile stimuli were presented using four electro-dynamic shakers attached to the surfaces of a custom-made chair. In this way the four shakers could excite the left arm, right arm, buttock, and back separately. The shakers could be driven by either the same signal in the mono condition or individual signals in the multi and incongruent conditions. Before presentation through the shakers, all signals were low-pass filtered in MATLAB using the *filter* function utilizing a 6th-order butterworth filter with a cutoff frequency of 200 Hz (even though human sensitivity for vibrations extends much higher^9^). The 200 Hz cutoff was determined via testing as a suited compromise between guaranteeing a broad-enough frequency response for driving vibration signals for all instrument classes on the one hand and minimizing acoustic sound radiation via the surfaces of the chair on the other.

The intensity of the vibration was adjusted as follows: Before Cohort 1 was tested, nine young pilot participants calibrated the vibration intensity of the chair to their ‘comfortable’ level using the mono condition. Four different songs were presented for the level adjustment, in which they could adjust the vibration intensity to the level they perceived as most comfortable. The presentation conditions differed between Cohort 1 and 2 in that the intensity calibration for Cohort 2 was done separately for each contact surface and for the mono and multi conditions. That is, before Cohort 2 was tested, again nine young pilot participants adjusted the left arm, right arm, back and the seating of the chair to the same felt level of intensity. The median of the adjusted levels were used in the subsequent main experiment. See Table S2 in the Supplementary Materials for the resulting absolute vibration levels.

### Apparatus

The experiment was conducted in a double-walled sound booth. Participants sat in the vibration chair for the entire duration of the experiment and interacted with a custom-made GUI displayed on a screen in front of them, which also guided them throughout the experiment. The presentation of the audio signals was processed through an *RME Fireface UFX II* soundcard at a sampling rate of 44.1 kHz and using the headphone output and open *Sennheiser HD 650* headphones. Vibrotactile stimulation was processed via 4 separate line outputs of the same audio interface and then directed to a *the t.racks DSP 4x4 mini amp* (4 × 50 W) amplifier. Each output signal of the amplifier was individually sent to one of the four shakers (*Rockwood bodyshaker*, 100 W, diameter of 120 mm, supplied via https://sintron-shop.de) were located at the back side of the wooden chair surfaces of the left arm rest, right arm rest, seat surface, and back rest. The shakers transmitted the signals to the respective chair parts, causing perceptible vibrations on the front side and the corresponding body part surfaces. Contact surfaces were decoupled from the chair frame and each other, see Fig. 1. The frequency responses of the individual surfaces are provided in Fig. S2 in the Supplementary Materials.

### Procedure

The research presented herein was approved by the ethics committee of the University of Oldenburg (Drs.EK/2019/092) and were performed in accordance with the relevant regulations as well as the Declaration of Helsinki. The data of this study were collected as part of a longer experiment with other distinct components that are not reported here. There were 32 trials overall (8 per vibration condition), presented in random order, which took participants on average around 30 min to complete. On every trial, participants were presented with excerpts of 30 s duration and were subsequently asked to provide ratings on seven-point Likert scales, based on the following questions (item titles in brackets):

- How pleasant is the effect of the music? (Valence)
- How energetic/arousing is the effect of the music? (Arousal)
- How much do you want to move to the groove of the music? (Groove)
- How much does this feel like being part of a live performance of the music? (LiveFeeling)
- How much do you have the feeling of being part of the music? (BeingPart)
- How much do you like the music? (Liking)

For the original questions in German language, the reader is referred to the Supplementary Materials.

### Supplementary Information

Supplementary Information.

## Data Availability

The datasets analyzed in the current study can be downloaded at https://github.com/Music-Perception-and-Processing/Vibro.
