# Supplementary material for: Vibrotactile enhancement of musical engagement
Source: Sci Rep. 2024 Apr 2;14:7764. doi: 10.1038/s41598-024-57961-8 (PMC10987628; doi:10.1038/s41598-024-57961-8)
Supplement: Supplementary file 1 — Supplementary Information. [file 41598_2024_57961_MOESM1_ESM.pdf]

Supplementary Materials
Vibrotactile enhancement of musical engagement

Kai Siedenburg[1,2,*], Michel Bürgel[2], Elif Özgür[2], Christoph Scheicht[2], and Stephan Töpken[2]

[1]Graz University of Technology, Signal Processing and Speech Communication Laboratory, 8010 Graz, Austria
[2]Carl von Ossietzy Universität Oldenburg, Dept. of Medical Physics and Acoustics, Oldenburg, D-26129 Germany
[*]kai.siedenburg@tugraz.at

ABSTRACT

Sound is sensed by the ear but can also be felt on the skin, by means of vibrotactile stimulation. However, only little research has addressed perceptual implications of vibrotactile stimulation in the realm of music. Here, we studied which perceptual dimensions of music listening are affected by vibrotactile stimulation and whether the spatial segregation of vibrations improves vibrotactile stimulation. Forty-one listeners were presented with vibrotactile stimuli via a chair's surfaces (left and right arm rests, back rest, seat) in addition to music presented over headphones. Vibrations were derived from different tracks of the music for each surface (multi condition) or conjointly by a mono-rendering, in addition to incongruent and headphones-only conditions. Listeners evaluated unknown music from popular genres according to valence, arousal, groove, the feeling of being part of a live performance, the feeling of being part of the music, and liking. Results indicated that the multi- and mono vibration conditions robustly enhanced the nature of the musical experience compared to headphones alone. Vibrotactile enhancement was strong in the latent dimension of 'musical engagement', encompassing the sense of being a part of the music, arousal, and groove. These findings highlight the potential of vibrotactile cues for creating intensive musical experiences.

Original formulations of test items in German language

- Wie angenehm ist der Effekt der Musik? (Valence)
- Wie energetisch/anregend ist der Effekt der Musik? (Arousal)
- Wie sehr haben Sie das Gefühl, Teil der Musik zu sein? (LiveFeeling)
- Wie sehr möchten Sie sich zum Groove der Musik bewegen? (Groove)
- Wie sehr haben Sie das Gefühl, dass die Musik Teil einer Live-Aufführung ist? (BeingPart)
- Wie sehr mögen Sie diese Musik? (Liking)

ID	Conditions	Artists	Songname	Genre	BPM	Start	End
1	Congruent	Dead Milkmen	Prisoners Cinema	Rock	135	661501	5953500
2	Congruent	Grants	Punch Drunk	Rap	100	661501	5953500
3	Congruent	Invisible Familiars	Disturbing Wildlife	Singer/Songwriter	80	3969001	9261000
4	Congruent	Midnight Blue	Hunting Season	Rock	200	4365901	9657900
5	Congruent	Mutual Benefit	Not For Nothing	Singer/Songwriter	125	882001	6174000
6	Congruent	Patrick Talbot	Fool	Singer/Songwriter	150	882001	6174000
7	Congruent	Secret Mountains	High Horse	Pop	125	1631701	6615000
8	Congruent	Strand of Oaks	Spacestation	Pop	85	1631701	6923700
9	Incongruent	Aimee Norwich	Child	Singer/Songwriter	145	2293201	7585200
10	Incongruent	Alexander Ross	Goodbye Bolero	Singer/Songwriter	165	441001	5733000
11	Incongruent	Auctioneer	Our Future Faces	Rock	85	1323001	6615000
12	Incongruent	Creepoid	Old Tree	Rock	130	1323001	6615000
13	Incongruent	Dahka Band	Soldier Man	World/Folk	125	1323001	6615000
14	Incongruent	Dreamers of the Ghetto	Heavy Love	Pop	120	2646001	7938000
15	Incongruent	Helado Negro	Mitad Del Mundo	Pop	120	1323001	6615000
16	Incongruent	Jade Safirah	Paraisso	Other	115	3969001	9261000

Table S1. Stimulus specifications. Songs were taken from the MedleyDB database [Bittner, R. M., Salamon, J., Tierney, M., Mauch, M., Cannam, C., and Bello, J. P. (2014). Medleydb: A multitrack dataset for annotation-intensive MIR research. In ISMIR, volume 14, pages 155–160.]. *Congruent* stimuli were used as acoustic stimuli in the headphones-only condition and also to drive the vibrotactile stimulation in the congruent playback conditions (Mono, Multi). Incongruent stimuli were used to drive the vibrotactile stimulation in the incongruent condition. The pairing between incongruent and congruent stimuli was randomized for each participant at the beginning of the experiment. BPM is an estimate of the beat rate (beats-per-minute) of the song. *Start* and *End* refer to the time point in samples that was used as the start and end to create the audio excerpt.

ID	Left Arm (Guitar)	Right Arm (Keys/Synth)	Back (Bass)	Seating (Drums)
1	125.8	131.3	112.4	110.1
2	130.7	130.4	113.4	112.7
3	129.5	130.2	110.3	110.5
4	129.4	128.2	110.1	112.2
5	132.6	133.0	109.8	113.5
6	131.3	129.9	119.4	112.3
7	129.8	131.2	114.9	112.1
8	129.5	130.2	112.1	109.9
9	130.2	131.7	113.1	111.2
10	129.0	118.1	114.7	113.4
11	126.4	131.7	110.1	110.7
12	126.9	124.5	113.5	111.0
13	129.9	127.8	117.6	111.2
14	131.6	131.1	116.3	112.4
15	129.1	131.6	112.0	110.7
16	108.0	120.2	99.4	116.2
avg.	129.5	130.1	114.0	112.2

Table S2. Measured vibration levels (dB re. $10^{-6} m/s^2$) at the different contact surfaces for the 16 songs (averaged across the 30 sec duration of the stimuli) after perceptual intensity equalization (before testing of Cohort 2), measured with a participant seated on the chair. The average across all 16 levels is displayed in the bottom row.

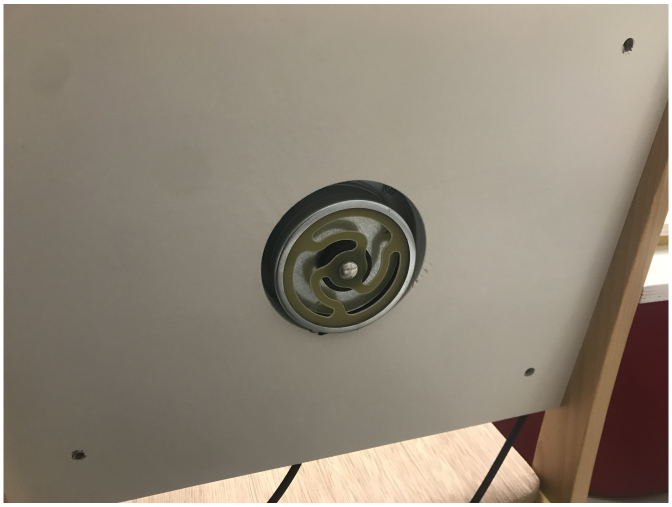

Figure S1. Electrodynamic shaker mounted to the back rest of the chair.

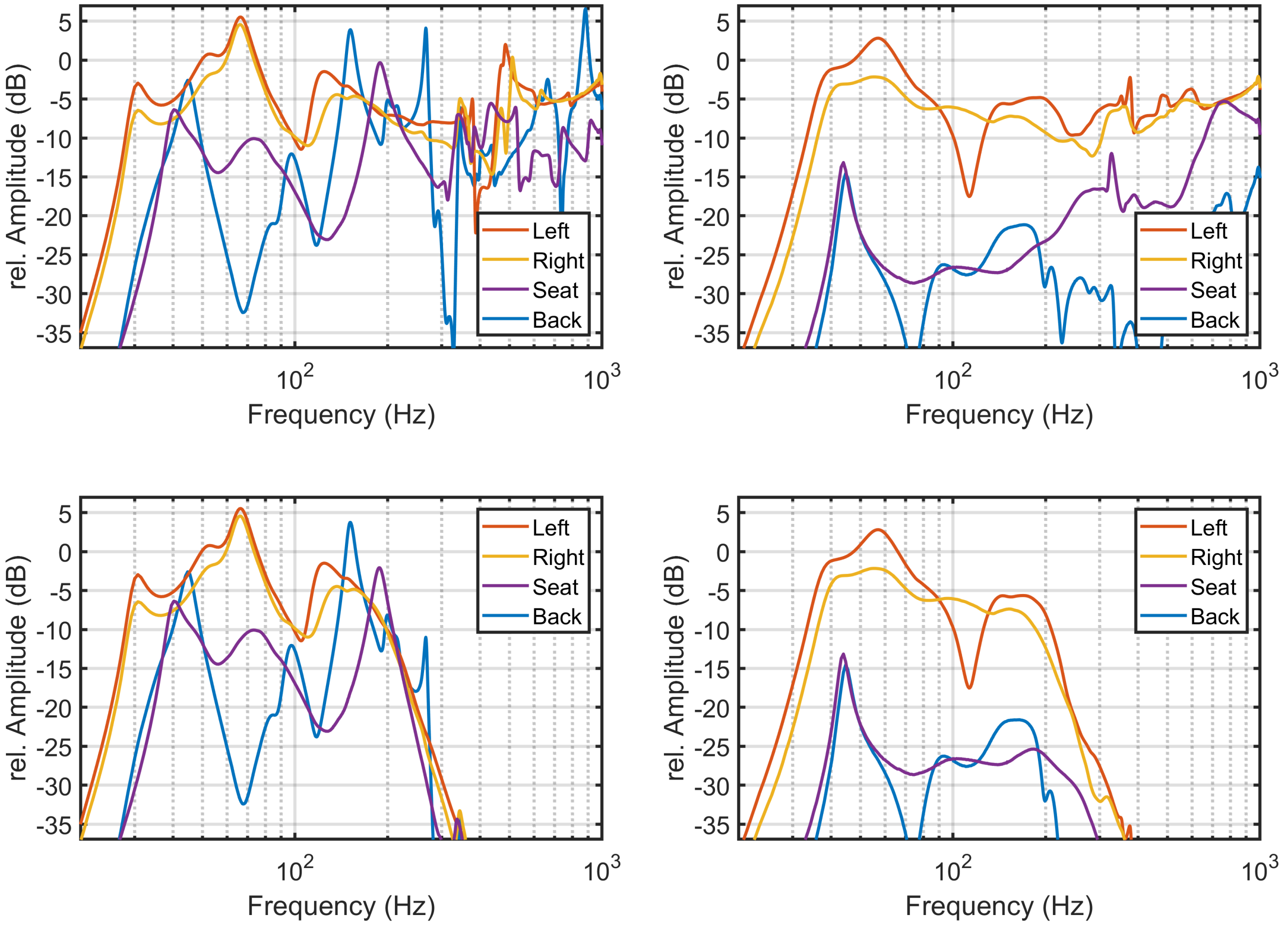

Figure S2. Relative amplitude transfer function for each vibration surface. Left column: response of empty chair. Right column: response with a participant (rather typical in terms of body height and weight) seated on the chair. Top row: raw measurements (up to 1000 Hz). Bottom row: measurement including the low-pass filter at 200 Hz, reflecting the effective frequency response that was valid in the stimulus presentation of the experiment.
